# Response to: “Merit of integrating in situ transcriptomics and anatomical information for cell annotation and lineage construction in single-cell analyses of *Populus*”

**DOI:** 10.1186/s13059-024-03228-4

**Published:** 2024-04-03

**Authors:** Shaoming Liang, Yiling Li, Yang Chen, Heng Huang, Sijia Li, Yuanzhong Jiang, Tao Ma

**Affiliations:** Key Laboratory of Bio-Resource and Eco-Environment of Ministry of Education, Sichuan Zoige Alpine Wetland Ecosystem National Observation and Research Station, College of Life Sciences, Sichuan University, Chengdu, China

## Main text

**Fig. 1:**
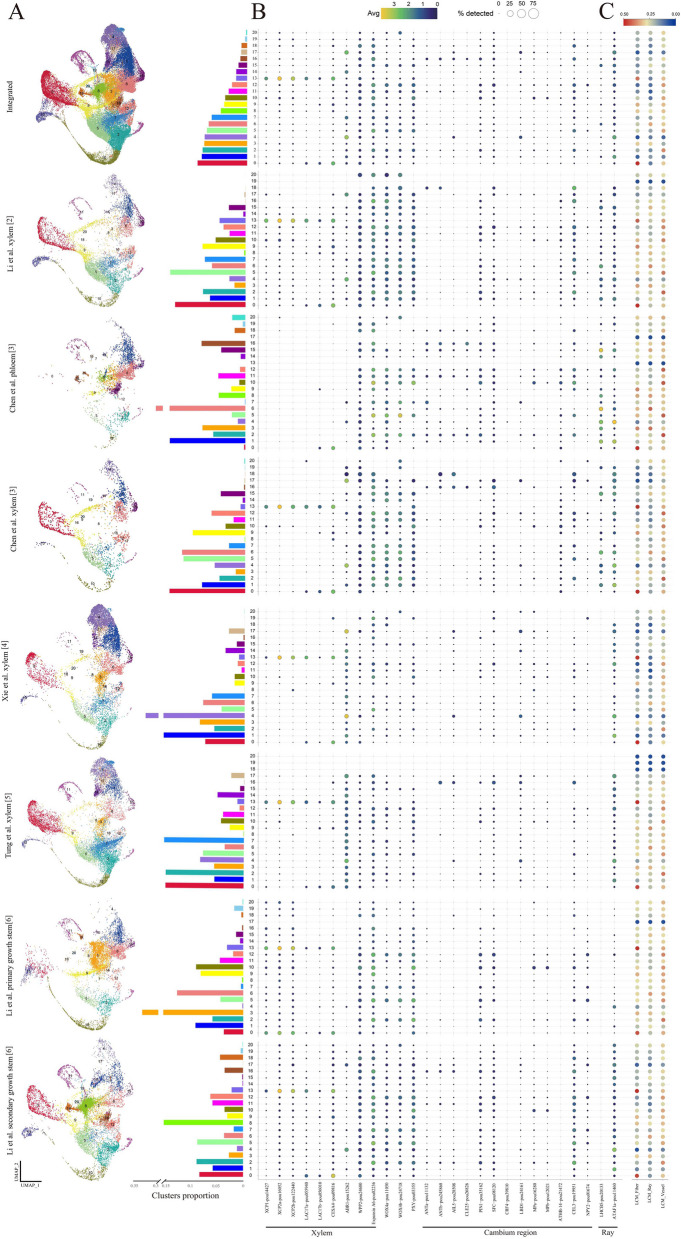
Single-cell atlas integrated based on 1:1 orthologous relationship. **A** UMAP visualization of different samples. Note that the data source is labeled to the left of the UMAP. Chen et al. [3] collected xylem and phloem cells separately, and Li et al. [6] collected primary growth stems and secondary growth stems separately, so we displayed these results separately. **B** The proportion of each cluster and the expression pattern of marker genes in different samples. **C** The correlation between each cluster and LCM-RNA-seq in different samples

We appreciate the correspondence from Chen et al. [1] regarding four recent studies on single-cell RNA-seq in poplar xylem [2–5]. Over the past 2 years, six research groups have investigated the cell types and differentiation trajectories in poplar woody tissues using single-cell and/or spatial transcriptome techniques [2–7], which provided valuable insights for future research on cambium development and secondary growth in tree species. However, as Chen et al. pointed out, there are discrepancies in the conclusions across these studies, particularly in terms of cell type identification and inferred pseudotime trajectories. To reach a consensus, it is essential to collect and organize all datasets from multiple sources along with other supporting evidence, such as in situ hybridization, transgenic or reporter lines in poplar, as well as in situ cellular transcriptome and anatomical information as suggested by Chen et al. [1]. In this response, we have gathered raw scRNA-seq data from five studies, excluding the study by Du et al. [7] due to the lack of slices containing spatial information in their data. Since different poplar species and reference genomes were employed in these studies, we integrated their expression matrices based on 1:1 orthologous relationship. This resulted in a final dataset comprising 62,256 cells, with a median of 1730 expressed orthologous genes, which can be divided into 21 cell clusters (Fig. 1A). Subsequently, we conducted a comprehensive analysis of the integrated dataset to try to elucidate the observed discrepancies.

First, we collated markers from these studies [8], and the integrated analysis indicates that plasticity in marker genes expression between samples may partially account for these discrepancies. For example, in two studies [2, 6], two genes related to normal programmed cell death, *XCP1* and *XCP2a*/*b*, were used as markers to identify vessel cells (Fig. 1B). After data integration, we found that these genes are specifically expressed in cell cluster 13 and showed high consistency across all samples. Similarly, the genes *LAC17* and *CESA4*, which were used for identifying xylem cells (including fibers &vessels) in another study [3], were specifically expressed in clusters 0 and 13 in all samples (Fig. 1B). These findings demonstrated the reliability and broad applicability of these markers in the identification of xylem cells. However, according to the AspWood database (plantgenie.org), XCPs may also be expressed in fiber cells, so there is still a lack of more marker genes to distinguish fiber and vessel cells. In comparison, the xylem parenchyma cell marker gene *ABR1*, identified by in situ hybridization in the study of Chen et al. [3], was mainly expressed in clusters 4 and 17 in all samples, except for one study where the gene was generally expressed in most cell types [5] (Fig. 1B). In particularly, the marker *WPP2* used for fiber identification in study of Xie et al. [4], and the candidate marker gene Expansin A6 for vessels in study of Tung et al. [5], did not show any cell type-specific expression patterns in almost all samples (Fig. 1B). These results indicate a wide diversity in the expression patterns of marker genes among different studies, which may be affected by factors such as seedling growth status, experimental procedures and differences between species, or more importantly, these genes may not be reliable as molecular markers for cell type identification. Nevertheless, it is undeniable that comparing multiple species and samples will be advantageous in identifying more conserved marker genes for achieving greater accuracy in cell identification in future research.

Second, integrative study confirms a significant level of cell heterogeneity in the cambium region. In line with the findings in the study of Li et al. [6], our results indicated that the previously recognized markers of cambium, *PXY* and *WOX4*, are extensively expressed in diverse cell clusters (Fig. 1B). We also found that the other markers used in these studies to identify cambium cells were not specifically expressed in any particular cell cluster (Fig. 1B). This suggests that the cells composing cambium may have strong cellular heterogeneity, or that these markers are also expressed in adjacent cells of cambium. Therefore, we recommend that these cells be referred to as “cambium region” or “cambium zone” until more definitive markers for cambium cells are established. Furthermore, we found that in the scRNA-seq data of debarked stems, the expression of cambium region marker genes such as *ANT* [9, 10] was observed (Fig. 1B). This suggests the possibility that cambium cells may be left on the wood side during bark peeling. Notably, the thickness of cell walls in tree stem tissue cells varies significantly, which can alter their proportions during protoplast preparation and lead to an inaccurate representation of the actual cellular composition of stem tissue in scRNA-seq data. In comparison, single-nuclei RNA-seq would cover a more comprehensive range of cell types and provide a more precise evaluation of cellular composition.

Third, correlation analysis shows that LCM (laser capture microdissection) combined with RNA-seq can provide another dimension of reference for cell classification, but the resolution is limited. In the study of Tung et al. [5], LCM-RNA-seq data for fiber, vessel, and ray cells were generated. We therefore conducted a correlation analysis between the expression levels of LCM-RNA-seq and the cell clusters identified here. The results showed that LCM-RNA-seq of fibers was closely related to the gene expression in cell clusters 0 and 13, which is generally consistent with the above results of cell annotation by xylem marker genes (Fig. 1C). Furthermore, LCM-RNA-seq of vessels revealed a similar correlation level with various cell types, including clusters 2, 5, 10, and 12 (Fig. 1C). However, there was not enough evidence to support the specific expression of genes related to secondary cell wall biosynthesis in these clusters. These results suggest that LCM-collected vessels may be a mixture of this cell type in multiple transcriptional states and that these clusters may represent vessels at early stages of development. Finally, in cell cluster 6, which had a slight correlation with the LCM-RNA-seq of ray cells (Fig. 1C), we did not find strong expression of the ray markers *LHCB5* and *ATAF1a*, which were validated by RNA in situ hybridization in two studies [2, 6]. This may be due to the significant heterogeneity of gene expression within anatomically defined cell types, and potential biases can be introduced when these cells are artificially combined and analyzed using RNA-seq, which requires further evaluation in the future. Therefore, this result also indicates that whether LCM can accurately distinguish various cell types in xylem remains questionable.

Taken together, our results demonstrate that there are still limitations in using existing techniques for xylem cell classification and subsequent analysis. In particular, identification of cell types based on a few marker genes may cause many conflicts. Therefore, it is still necessary to utilize more xylem single-cell data from different species and developmental stages to mine more reliable and conserved marker genes. In addition, the combination of single-cell, single-nuclei RNA-seq, spatial data, and more refined LCM analysis will also bring us more discoveries. It is believed that with the advancement of technology and the accumulation of relevant evidence, our understanding of woody plants will be significantly improved in the future.

### Supplementary Information

**Additional file1.** Review history.

## Data Availability

The data were downloaded from GenBank (PRJNA703312 [2] and PRJNA746591 [5]) and the National Genomics Data Center (PRJCA005543 [3], PRJCA014789 [4] and PRJCA015012 [6]). The single-cell expression matrix integrated is available on Zenodo [11].
