# Supplementary material for: Response to: “Merit of integrating in situ transcriptomics and anatomical information for cell annotation and lineage construction in single-cell analyses of Populus”
Source: Genome Biol. 2024 Apr 3;25:86. doi: 10.1186/s13059-024-03228-4 (PMC10988792; doi:10.1186/s13059-024-03228-4)
Supplement: Supplementary file 1 — Additional file1. Review history. [file 13059_2024_3228_MOESM1_ESM.docx]

Review History

**First round of review**

**Reviewer 1**

**Were you able to assess all statistics in the manuscript, including the appropriateness of statistical tests used?**

**There are no statistics in the manuscript.**

**Comments to author:**

This is a response to the commentary by Chen et al., 2023 where certain criticism was raised to the original work (Chen et al., 2021) from the Ma laboratory. The response raises important points on the technology of poplar wood scRNaseq used in several publication, including 1) the enigmatic expression of cambial marker genes and 2) the fact that peeling of the tree bark, as performed typically in the wood scRNAseq analyses, does not necessarily exclude cambial cells. The latter discussion is a direct response to the criticism raised in the commentary by Chen et al., 2023 as well in their original work (Tung et al., 2023) on the methodology and results in Chen et al., 2021. My view is that the authors are correct in their description of the technological challenges when collecting the cambial region after peeling of the bark.

The response contains also integration of all scRNAseq data available to date on poplar stem tissues. This is a valuable addition to the existing analyses. One part of the analysis focuses on comparison of the de novo scRNAseq data clustering to LCM data from Tung et al., 2023. This comparison is done to validate the analyses and conclusion "the model proposed by Tung et al 2023 would be the most plausible one, on the basis of the available scientific data, for explaining differentiating xylem development", reported in the commentary by Chen et al., 2023. The analysis of the current report indeed challenges this conclusion, but contains also some caveats, as listed below.

(i) the LCM fiber sample (from Tung et al., 2023) correlates with two scRNAseq clusters; 0 and 13. Cluster 0 is not commented here further but from line 81 one gets the impression that the authors consider the correlation with cluster 13 problematic. Is this since the authors consider cluster 13 as vessels? In that case it is premature. The evidence for cluster 13 being vessels is suggested by the expression of XCP1/2 specifically in cluster 13. However, XCPs are likely to be expressed also in fibers on the basis of for instance the AspWood database (plantgenie.org).

(ii) the LCM vessel sample correlates with many different clusters. This is however shown already in Tung et 2021. Also Chen et al. 2023 reported the vessel LCM sample correlating not only with the vessel marker genes but also fiber and ray markers (figure 1I in Chen et al. 2023). And in the current analysis, we cannot, as pointed out above, be sure that cluster 13 only consists of vessels. In addition, cells in clusters 2 and 10 could be considered as earlier stages of cells in cluster 13 (vessels ?), which suggests that the LCM vessel sample, perhaps together with a minor proportion of other cell types, could consists of vessels in earlier stages of differentiation than the cells in cluster 13 . I urge the authors to clarify the discussion concerning the enigmatic vessel LCM sample from Tung et al. 2013.

(iii) The authors question the identify of the LCM ray sample on the basis of two genes that have been identified as marker genes in their own, earlier work. It does not become clear why the authors consider these particular genes as the most reliable ray markers. Some more clarification on this matter would be good.

Taken together, I urge the authors to enlighten the various aspects that influence interpretation of the scRNA and LCM results, and how the community could continue from here 'to reach a consensus'. Some of the important steps could include 1) identification of sets of marker genes rather than single genes, 2) combination with spatial transcriptomics, 3) more elaborate LCM analyses.

Lines 36-37. Curiously, Tung et al. 2023 reports in Additional file 17 that XCP2 is enriched in libriform fibers in the scRNAseq results, but in vessel elements in the lcmRNAseq results.

Lines 48-51. Isn't the correct conclusion rather that these genes are not good marker genes?

Line 25. It is not clear what is meant by " due to the lack of spatial information in their data". Du et al., provides spatial data but not their own scRNAseq data. Do you mean "due to the lack of scRNAseq data".

Lines 44-45." except for one study where the gene was generally expressed in most cell types [5]". It seems, even though it is difficult to see the exact colours in the figure 1, that the correlation is best anyway with clusters 4 and 17. Please specify.

Lines 48-51. It is not very likely that the different species or genotypes would have contributed to wide diversity in the expression patterns of marker genes or the cluster identity altogether.

Line 81. What is meant by "roughly consistent with above cell annotations". Could you be a bit more precise ?

References

Chen Y-L,Hsieh J-W A, Kuo S-C , Kao C-T , Tung C-C , Yu J-H , Chang T-S , Ku C , Xie J, Zhang D, Li Q , Lin YJ. 2023. Merit of integrating in situ transcriptomics and anatomical information for precision cell annotation and lineage construction in singlecell analyses. Genome Biology in press

Chen Y, Tong S, Jiang Y, Ai F, Feng Y, Zhang J, Gong J, Qin J, Zhang Y, Zju Y, Liu H, Ma T. 2021. Transcriptional landscape of highly lignified poplar stems at single-cell resolution. Genome Biol. 22:319.

Tung CC, Kuo SC, Yang CL, Yu JH, Huang CE, Liou PC, Sun YH, Shuai P, Su JC, Ku C, Lin YJ. 2023. Single-cell transcriptomics unveils xylem cell development and evolution. Genome Biol. 24:3.

**Authors Response**

**Point-by-point responses to the reviewers’ comments:**

Reviewer #1: This is a response to the commentary by Chen et al., 2023 where certain criticism was raised to the original work (Chen et al., 2021) from the Ma laboratory. The response raises important points on the technology of poplar wood scRNaseq used in several publication, including 1) the enigmatic expression of cambial marker genes and 2) the fact that peeling of the tree bark, as performed typically in the wood scRNAseq analyses, does not necessarily exclude cambial cells. The latter discussion is a direct response to the criticism raised in the commentary by Chen et al., 2023 as well in their original work (Tung et al., 2023) on the methodology and results in Chen et al., 2021. My view is that the authors are correct in their description of the technological challenges when collecting the cambial region after peeling of the bark.

*Reply: We appreciate the reviewer’s positive comments on our work.*

(i) the LCM fiber sample (from Tung et al., 2023) correlates with two scRNAseq clusters; 0 and 13. Cluster 0 is not commented here further but from line 81 one gets the impression that the authors consider the correlation with cluster 13 problematic. Is this since the authors consider cluster 13 as vessels? In that case it is premature. The evidence for cluster 13 being vessels is suggested by the expression of XCP1/2 specifically in cluster 13. However, XCPs are likely to be expressed also in fibers on the basis of for instance the AspWood database (plantgenie.org).

*Reply: Thank you for pointing this out. Since XCP1/2 may also be expressed in fibers according to the AspWood database (plantgenie.org) you suggested, we deleted the sentence ‘However, it could not explain the specific expression of vessel markers in cluster 13’ to avoid controversy’. We also added the description of the expression pattern of XCPs in the revised manuscript at lines 42-44.*

(ii) the LCM vessel sample correlates with many different clusters. This is however shown already in Tung et 2021. Also Chen et al. 2023 reported the vessel LCM sample correlating not only with the vessel marker genes but also fiber and ray markers (figure 1I in Chen et al. 2023). And in the current analysis, we cannot, as pointed out above, be sure that cluster 13 only consists of vessels. In addition, cells in clusters 2 and 10 could be considered as earlier stages of cells in cluster 13 (vessels ?), which suggests that the LCM vessel sample, perhaps together with a minor proportion of other cell types, could consists of vessels in earlier stages of differentiation than the cells in cluster 13. I urge the authors to clarify the discussion concerning the enigmatic vessel LCM sample from Tung et al. 2013.

*Reply: We agree with you that xylem cells are extremely heterogeneous. Although there is insufficient evidence to support specific expression of genes related to secondary cell wall biosynthesis in these clusters, we cannot rule out the possibility that they may be vessels at an early stage of development. Based on your suggestion, we revised this description in lines 87-90 of the article, retaining the possibility that these clusters are vessel cells in early stages of development.*

(iii) The authors question the identify of the LCM ray sample on the basis of two genes that have been identified as marker genes in their own, earlier work. It does not become clear why the authors consider these particular genes as the most reliable ray markers. Some more clarification on this matter would be good.

*Reply: We do not consider these two genes to be reliable molecular markers for identifying ray cells, we simply want to show that there is a conflict in annotating ray cells using LCM-RNA-seq and marker genes. Therefore, it is still questionable whether LCM can accurately distinguish ray cells or whether scRNA-seq data can accurately capture rays, and further more detailed research is needed. Additionally, as you mentioned, more molecular markers are needed to distinguish the expression signatures of these cells and their inherent heterogeneity. This is also why further use of spatial transcriptome, scRNA-seq, snRNA-seq and more elaborate LCM analysis is necessary. We have added these discussions in lines 100-108 at the end of this article.*

Taken together, I urge the authors to enlighten the various aspects that influence interpretation of the scRNA and LCM results, and how the community could continue from here 'to reach a consensus'. Some of the important steps could include 1) identification of sets of marker genes rather than single genes, 2) combination with spatial transcriptomics, 3) more elaborate LCM analyses.

*Reply: Thanks for your very valuable suggestions, we have added these discussions in lines 100-108 at the end of this article.*

Lines 36-37. Curiously, Tung et al. 2023 reports in Additional file 17 that XCP2 is enriched in libriform fibers in the scRNAseq results, but in vessel elements in the lcmRNAseq results.

*Reply: Thank you for pointing this out. As you mentioned, we also found that the XCP2 gene exhibits conflicting results in scRNA-seq and LCM results. However, when we integrated all scRNA-seq data, we found that this gene has very specific expression in cell cluster 13. Therefore, this conflict may be caused by errors in LCM cell type identification, or as you mentioned, LCM may be mixed by cells in various states.*

Lines 48-51. Isn't the correct conclusion rather that these genes are not good marker genes?

*Reply: We strongly agree with your view that the diversity of expression patterns of these genes in multiple studies illustrates the unreliability of these genes as molecular markers to identify cell types. So we have revised the description to ‘These results indicate a wide diversity in the expression patterns of marker genes among different studies, which may be affected by factors such as seedling growth status, experimental procedures and differences between species, or more importantly, these genes may not be reliable as molecular markers for cell type identification’.*

Line 25. It is not clear what is meant by " due to the lack of spatial information in their data". Du et al., provides spatial data but not their own scRNAseq data. Do you mean "due to the lack of scRNAseq data".

*Reply: The data uploaded by Du et al. lack slices containing spatial information, which hinders our processing of the original data. To avoid ambiguity, we provide further clarification at line 25 of the revised manuscript.*

Lines 44-45." except for one study where the gene was generally expressed in most cell types [5]". It seems, even though it is difficult to see the exact colours in the figure 1, that the correlation is best anyway with clusters 4 and 17. Please specify.

*Reply: The size of the circle in Figure 1B represents the proportion of cells expressing a certain gene in each cluster. In the data of Tung et al., the expression of ABR1 can be detected in every cluster and the proportion of cells is relatively high. In other studies, except for clusters 4 and 17, the expression proportion of this gene in other clusters is very low. This result indicates that some molecular markers are not conserved among these studies. We have added relevant discussion to this section of the article at lines 97-98.*

Lines 48-51. It is not very likely that the different species or genotypes would have contributed to wide diversity in the expression patterns of marker genes or the cluster identity altogether.

*Reply: We have revised the description to ‘These results indicate a wide diversity in the expression patterns of marker genes among different studies, which may be affected by factors such as seedling growth status, experimental procedures and differences between species, or more importantly, these genes may not be reliable as molecular markers for cell type identification’.*

Line 81. What is meant by "roughly consistent with above cell annotations". Could you be a bit more precise ?

*Reply: We have modified it to ‘which is generally consistent with the above results of cell annotation by xylem marker genes’ at Lines 84-85 of the revised manuscript.*
